# The Characteristics of Appendicoliths Associated with Acute Appendicitis

**DOI:** 10.7759/cureus.5322

**Published:** 2019-08-05

**Authors:** Muhammad Sohaib Khan, Mustafa Belal Hafeez Chaudhry, Noman Shahzad, Muhammad S Khan, Maryiam Wajid, Wasim A Memon, Rehman Alvi

**Affiliations:** 1 Surgery, Aga Khan University, Karachi, PAK; 2 Radiology, Aga Khan University, Karachi, PAK; 3 General Surgery, East Kent Hospitals University National Health Service Foundation Trust, Margate, GBR; 4 Global Health Heal Initiative, University of California San Francisco, San Francisco, USA; 5 Radiology, Aga Khan University Hospital, Karachi, PAK

**Keywords:** appendicoliths, acute appendicitis, incidental, computed tomography

## Abstract

Introduction

Differences between appendicoliths associated with appendicitis and those found incidentally have not been studied. The objective of this study was to determine the characteristics of appendicoliths that are associated with acute appendicitis.

Methods

A cross-sectional study of patients with appendicoliths identified on computed tomographic (CT) scan from January 2008 till December 2014 was conducted. Patients were divided into two group: appendicitis and appendicoliths (AA) and incidentally discovered appendicoliths (IA).

Results

Overall, 321 patients were included in the study. Of these, 103 (32%) patients were in the AA group while 218 (68%) patients were in the IA group. Both groups were similar in age and gender distribution. Significantly greater proportion of patients in the AA group had more than one appendicolith [AA vs. IA: 63 (62%) vs. 82 (38%), p < 0.001], appendicolith location at the base [AA vs. IA: 34 (33%) vs. 33 (15%), p < 0.001] and appendicolith diameter of 5 mm or more [AA vs. IA: 71 (69%) vs. 28 (13%), p < 0.001]. On multivariate analysis, more than one appendicolith [Odds ratio (OR): 1.9, 95% CI: 1.1-3.4; p = 0.02] and diameter of 5 mm or more (OR: 13, 95% CI: 7.1-23.6; p < 0.001) were independently associated with acute appendicitis.

Conclusion

Multiple appendicoliths and appendicoliths larger than 5 mm are associated with acute appendicitis.

## Introduction

Acute appendicitis is one of the most common acute abdominal emergencies with an incidence ranging from 100 to 200 cases per 100,000 person years [1]. Understanding pathogenesis and identifying etiological factors is crucial for prevention, early diagnosis and better management of this common condition. While multiple etiological factors have been identified, the most widely reported and accepted mechanism is the obstruction of appendiceal lumen by an appendicolith resulting in inflammation [2]. Wangensteen and Dennis first demonstrated this experimentally in 1939 [3]. Since then numerous studies have shown appendicoliths to be the most common and important obstructive factor [2, 4]. Recently, appendicoliths were reported to be significantly associated with appendiceal perforation and gangrene as well [5, 6]. The mechanism behind certain appendicolith causing appendicitis remains unclear. Appendicoliths are found incidentally in up to 32% of asymptomatic population [7]. A recent study reported that none of the patients who had incidentally discovered appendicoliths developed acute appendicitis during the follow-up period of four years [8].

Therefore, the objective was to study the characteristics of the appendicoliths associated with the development of acute appendicitis.

## Materials and methods

This was a retrospective cross-sectional study of patients identified with appendicoliths on computed tomographic (CT) scan from January 2008 to December 2014. All patients who were 16 years or older of age at the time of the study were included. The electronic radiology database was queried using the term “Appendicolith” to search for CT scans that reported presence of appendicolith. These were reviewed for the purpose of this study by a radiologist. Appendicoliths were said to be present when an appendiceal intraluminal high attenuation focus was observed on review. Institutional ethics review committee approval was obtained prior to commencement of the study.

The patients were divided on the basis of radiological evidence of appendicitis as either “Appendicitis with Appendicoliths (AA)” or “Incidental Appendicolith without appendicitis (IA)”. Radiological diagnosis of appendicitis was made when appendiceal dilatation was of >6 mm with any of the following additional features were present: appendiceal wall thickness of >3 mm, peri-appendiceal fat stranding, peri-appendiceal free fluid or heterogenous appendiceal wall enhancement (in case of contrast-enhanced CT imaging). Those who had appendicoliths but did not have any radiological evidence of appendicitis were placed in the IA group. Variables that were collected included age, gender, the number, position and diameter of appendicoliths as assessed from the CT scans.

The variables were categorized based on the diameter of the appendicolith (<5 mm or ≥5 mm) and the location of the appendicolith (base or distally) similar to an earlier reported study [6]. Categories for the number of appendicoliths were made as single appendicolith or >1 appendicolith.

Statistical analysis

Categorical variables are reported as frequencies with percentages, while continuous variables are reported as means with two standard deviations. Chi-square test was used as the test of significance to detect difference between two groups for categorical variables, while Independent Samples t-test was used for continuous variables. Variables with p < 0.2 at univatriate analysis were included in the multivariable logistic regression analysis. P-value <0.05 was considered significant. Statistical analysis was performed using Statistical Package for Social Sciences (SPSS) version 19 (IBM Corp., Armonk, NY).

## Results

**Figure 1:**
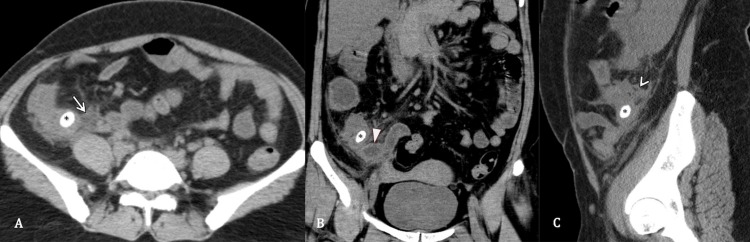
Appendicitis and appendicolith Contrast-enhanced CT abdomen, axial section (A), coronal section (B) and sagittal section (C), of a 38-year-old female patient, presented with abdominal pain and anorexia. CT shows an impacted appendicolith at the base of the appendix (asterisk) with a streak of periappendiceal fluid (arrow), dilated appendix (solid arrow head) and diffuse periappendiceal inflammation (hollow arrowhead).

**Figure 2:**
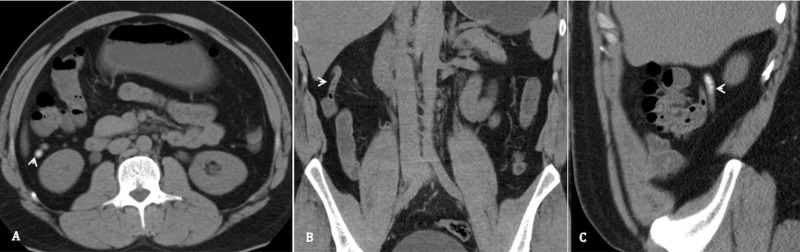
Incidental appendicolith Unenhanced CT abdomen, axial section (A), coronal section (B) and sagittal section (C), of a 23-year-old female with left lumbar pain. It is demonstrating a non-inflamed appendix (arrow head) with hyperattenuating tiny appendicoliths in its lumen.

In total, 321 patients were found to have appendicoliths on abdominal CT scans. Mean age was 36 ± 15.6 years with 103 (32%) females. Of these, 103 (32%) patients had acute appendicitis (Figure 1) and were included in the AA group and 218 (68%) patients had incidental appendicoliths (Figure 2) and were included in the IA group. There was no statistically significant difference in the age or gender distribution between the two groups (Table 1).

**Table 1:** Patient characteristics *Appendicoliths with appendicitis **Incidental appendicoliths ***Standard deviation

	AA* n = 103 (32%)	IA** n = 218 (68%)	p-value
Mean Age ± SD*** (years)	34 +- 14	37 +- 16	0.102
Females (%)	27 (26.2)	76 (73.8)	0.121

On univariate analysis, significantly greater proportion of patients in the AA group were found to have: >1 appendicolith [AA vs. IA: 63 (62%) vs. 82 (38%); p < 0.001], location of largest appendicolith at the base [AA vs. IA: 34 (33%) vs. 33 (15%); p < 0.001] and diameter of the largest appendicolith ≥5 mm [AA vs. IA: 71 (69%) vs. 28(13%); p < 0.001] (Table 2).

**Table 2:** Appendicolith characteristics *Appendicoliths with appendicitis **Incidental appendicoliths

	AA* n = 103 (32%)	IA** n = 218 (68%)	p-value
Size			
Less than 5 mm	32 (31)	190 (87)	<0.001
5 mm or more	71 (69)	28 (13)	
Number			
1 or Sludge	40 (38)	136 (62)	<0.001
More than 1	63 (62)	82 (38)	
Position			
Base	34 (33)	33 (15)	<0.001
Distally	69 (67)	185 (85)	

On multivariable logistic regression analysis, >1 appendicolith [OR: 1.9, 95% Confidence Interval (CI): 1.1-3.4; p = 0.02)] and diameter of largest appendicolith ≥5 mm [OR: 13, 95% CI: 7.1-23.6; p < 0.001] were found to be associated with acute appendicitis (Table 3).

**Table 3:** Multivariable logistic regression analysis

Covariates	Odds Ratio (OR)	p-value	95% Confidence Interval (CI)	
Age	1.01	0.12	0.9	1.0
Gender	0.58	0.58	0.3	1.1
Location Base	0.94	0.55	0.27	1.1
Size of 5 mm or more	12.97	<0.00	7.12	23.6
More than 1 Appendicolith	1.9	0.02	1.07	3.47

## Discussion

Our results show that appendicoliths that are larger in size and multiple in number are associated with acute appendicitis. These results add to the understanding of the pathogenesis of acute appendicitis and will aid clinicians to take appropriate management decisions in equivocal presentations. The hypothesis of luminal obstruction being the primary triggering factor of acute appendicitis, demonstrated first by Wangensteen and Dennis in 1939, is endorsed by our findings. This concept suggests that the obstruction by appendicolith leads to pooling of secretions within the appendiceal lumen, hence, lead to increased intraluminal pressure, venous congestion and proliferation of bacteria [3]. These changes collectively result in an inflammatory reaction within the appendix. Therefore, larger appendicoliths and multiple appendicoliths that may cause multilevel luminal obstructions are more likely to cause acute appendicitis.

Appendicoliths have also been shown to exist in asymptomatic population. Jones et al. described the presence of asymptomatic appendicoliths in up to 32% of Canadian patients by intraoperatively palpating the appendices during non-appendiceal surgeries [7]. These incidentally discovered appendicoliths have not been found to increase the risk of appendicitis. In a recently published study that included 111 patients identified with asymptomatic appendicoliths on CT scan, none of the patients developed appendicitis at a mean follow-up of four years [8]. Other factors have also been found to be associated with the development of acute appendicitis. These include infectious agents, genetic and familial factors, and monthly variation in climate, air pollution, and smoking [9-13]. However, appendicoliths continue to be the most frequently reported etiological factor [2, 4]. Why certain appendicoliths tend to cause appendicitis while others do not is not known. This study has attempted to answer this question by identifying radiological characteristics of appendicoliths associated with appendicitis.

Larger size of appendicoliths and its more proximal location has also been found to be associated with complicated appendicitis [6]. Perforations may occur due to high luminal pressure from ongoing obstruction leading to ischemia, gangrene and ultimately rupture of the appendix [6]. These findings have been confirmed in pediatric age group as well [5]. Khan et al. recently described that odds of having appendicoliths was 2.4 times greater for patients with complicated appendicitis [14]. Therefore, while interpreting the CT scans of patients suspected to have acute appendicitis, consideration should be given to the size and number of appendicoliths. If present, these should increase the suspicion of acute appendicitis, especially in equivocal cases.

This study is first of its kind to look into specific radiological characteristics of appendicoliths associated with acute appendicitis. However, there are certain limitations. This study is based upon retrospectively collected hospital data. Moreover, clinical features and laboratory parameters have not been included. Our findings therefore need to be revalidated through prospective studies that may also include long-term follow-up of asymptomatic patients who have large and multiple appendicoliths.

## Conclusions

In conclusion, appendicoliths that measure 5 mm or more, or are multiple are associated with acute appendicitis. High clinical suspicion must be observed for patients who have equivocal clinical signs and symptoms for acute appendicitis but have appendicoliths with these characteristics.

## References

[1] Ferris M, Quan S, Kaplan BS (2017). The global incidence of appendicitis: a systematic review of population-based studies. Ann Surg.

[2] Ramdass MJ, Young Sing Q, Milne D, Mooteeram J, Barrow S (2014). Association between the appendix and the fecalith in adults. Can J Surg.

[3] Wangensteen OH, Dennis C (1939). Experimental proof of the obstructive origin of appendicitis in man. Ann Surg.

[4] Forbes GB, Lloyd-Davies RW (1966). Calculous disease of the vermiform appendix. Gut.

[5] Alaedeen DI, Cook M, Chwals WJ (2008). Appendiceal fecalith is associated with early perforation in pediatric patients. J Pediatr Surg.

[6] Ishiyama M, Yanase F, Taketa T, Makidono A, Suzuki K, Omata F, Saida Y (2013). Significance of size and location of appendicoliths as exacerbating factor of acute appendicitis. Emerg Radiol.

[7] Jones BA, Demetriades D, Segal I, Burkitt DP (1985). The prevalence of appendiceal fecaliths in patients with and without appendicitis. A comparative study from Canada and South Africa. Ann Surg.

[8] Khan MS, Chaudhry MBH, Shahzad N, Tariq M, Memon WA, Alvi AR (2018). Risk of appendicitis in patients with incidentally discovered appendicoliths. J Surg Res.

[9] Lamps LW (2010). Infectious causes of appendicitis. Infect Dis Clin North Am.

[10] Ergul E (2007). Heredity and familial tendency of acute appendicitis. Scand J Surg.

[11] Wei PL, Chen CS, Keller JJ, Lin HC (2012). Monthly variation in acute appendicitis incidence: a 10-year nationwide population-based study. J Surg Res.

[12] Kaplan GG, Dixon E, Panaccione R (2009). Effect of ambient air pollution on the incidence of appendicitis. CMAJ.

[13] Oldmeadow C, Wood I, Mengersen K, Visscher PM, Martin NG, Duffy DL (2008). Investigation of the relationship between smoking and appendicitis in Australian twins. Ann Epidemiol.

[14] Khan MS, Siddiqui MTH, Shahzad N, Haider A, Chaudhry MBH, Alvi AR (2019). Factors associated with complicated appendicitis: view from a low-middle income country. Cureus.

